# What motivates food workers to adhere with WHO guidelines to combat the COVID-19? A social cognitive theory perspective

**DOI:** 10.3389/fpubh.2023.1187056

**Published:** 2023-10-19

**Authors:** Mingliang Wang, Shunlong Gong, Jin Tang, Zhenlin Weng, Xingtong Wu

**Affiliations:** ^1^School of Business and Management, Jilin University, Changchun, China; ^2^Jiangxi Rural Revitalization Strategy Research Institute, Jiangxi Agricultural University, Nanchang, China

**Keywords:** self-efficacy, risk perception, food safety, hygiene practices, foodborne illnesses, job stress

## Abstract

**Background:**

COVID-19 has become a public health emergency and pandemic of global concern, and the hundreds of millions of foodborne illnesses that occur each year also wreak havoc on human lives, society and the economy. Promoting workers in food service establishments to adhere to the hygiene practices in the WHO guidelines is a two-birds-one-stone strategy in preventing the spread of COVID-19 and limiting the occurrence of foodborne illness. The aim of this study was to determine the drivers that motivate workers to adhere to hygiene practices based on social cognitive theory.

**Methods:**

The cross-sectional survey targeting food workers using face-to-face interviews was conducted from July to September 2022. Stratified random sampling and convenience sampling were employed to locate survey sites and respondents, respectively. The survey uses a credible questionnaire evaluated by multiple reliability and validity measures. Binary logistic regression was employed to identify significant determinants of adherence to WHO-initiated hygiene practices.

**Results:**

A total of 900 workers were interviewed and 609 valid questionnaires were received. The study showed that the average correct rate of knowledge about hygiene practices was only 51.09%, that perceived non-adherence to hygiene practices was most likely to result in lower customer satisfaction and the spread of COVID-19, and that only about 11.7% of the workers always adhered to hygiene practices. Three of the cognitive dimensions in the personal factors, self-efficacy, risk perception, and knowledge, had significant positive effects on adherence practices. Among the demographic variables, there were significant differences in adherence practices differing by income level and place of residence.

**Conclusion:**

It was found that workers’ knowledge of the WHO-initiated hygiene practices is insufficient and that the frequency of adherence to hygiene practices is poor and require improvement. The significant drivers and effects of demographic variables provide evidence-based guidance to identify priority intervention information and populations to improve worker hygiene practices.

## 1. Introduction

Since it was first reported in November 2019, Coronavirus Disease 2019 (COVID-19) has rapidly spread worldwide, with over 700 million confirmed cases and more than 6.8 million deaths by February 2023 (WHO, 2023), and has been recognized as a public health emergency and pandemic of global worry (1). COVID-19 is not a foodborne disease and is principally transmitted from person to person through direct or indirect exposure. But anything that surrounds food, like food packaging, utensils, and tabletop, may still be a vehicle or petri dish for the spread of the virus (2). It has been accepted that improper handling of food may lead to infection with the virus (3–5). Food service establishments have become the worst-hit by the transmission of the virus (6). WHO has issued official guidelines, *COVID-19 and food safety*, for the sake of standardize the behavior of workers in the food business to prevent the spread of COVID-19, covering two main areas: wearing personal protective equipment and good hygiene practices (7). Notably, good hygiene practices are not only effective in preventing the spread of COVID-19, but also one of the key measures in reducing the occurrence of foodborne diseases (8, 9). Furthermore, motivating food workers adherence the hygiene practices in the WHO guidelines is important to restore consumer trust and market activity under the ravages of COVID-19.

In order to promote adherence to WHO guidelines by food workers, it is necessary to identify the drivers. Prior to the COVID-19 outbreak, several scholars have attempted to understand the influencing factors of food handling behaviors of food workers (10–12). Relevant studies after the outbreak, however, are limited, especially the lack of studies on Chinese food workers. Existing research is mostly focused on the effects of knowledge, attitudes, and demographic variables (5, 13, 14). However, many studies have confirmed that while knowledge and attitude exert influence on behavior formation, it has also been determined that individuals with knowledge and positive attitudes do not necessarily act accordingly, i.e., there exists knowledge–behavior gap and attitude-behavior gap (15, 16). It is suggested that the applying social psychological theories involving individual thought processes and social influences enables better understanding of the behavior formation mechanisms (17).

Social cognitive theory (SCT), proposed by Bandura (18), is one of the most influential theories for explaining human behavior, and has been successfully adopted to explain health behavior, information-seeking behavior, pro-environmental behavior (19–21). However, there is no literature that uses SCT to explore the determinants of food workers’ adherence to WHO guidelines, including good hygiene practices, against COVID-19 until now. In view of the existing research gap, this study is the first attempt to apply SCT to identify the determinants of adherence to WHO-initiated hygiene practices. SCT holds that human behavior is dominated by ternary reciprocal causality and that behavioral, environmental, and personal factors interact and influence each other in a reciprocal deterministic framework (22). In accordance with this framework, personal factors were attempted to be constituted in two dimensions: cognitive factors and affective factors. The cognitive factors involved knowledge, self-efficacy, and risk perception, and the affective factor refers to the job stress of food workers. Environmental factors in this study refer to facilities adequacy in the working site. Furthermore, this was confirmed that there were significant differences between food workers’ practices under different demographic variables (5, 13). Thus, the effects of demographic variables were examined to better understand the drivers of adherence to WHO-initiated hygiene practices.

In conclusion, the aim of this study based on a SCT perspective was designed to explore two questions: the current status of food workers’ knowledge, risk perception, facilities adequacy, and adherence to WHO-initiated hygiene practices, and their job stress in China; examining the effects of personal factors (both cognitive factors and affective factors), environmental factors, and demographic variables on worker adherence practices. From these results, theoretical guidance was established for the design of a practice intervention for food workers.

## 2. Materials and methods

**Figure 1:**
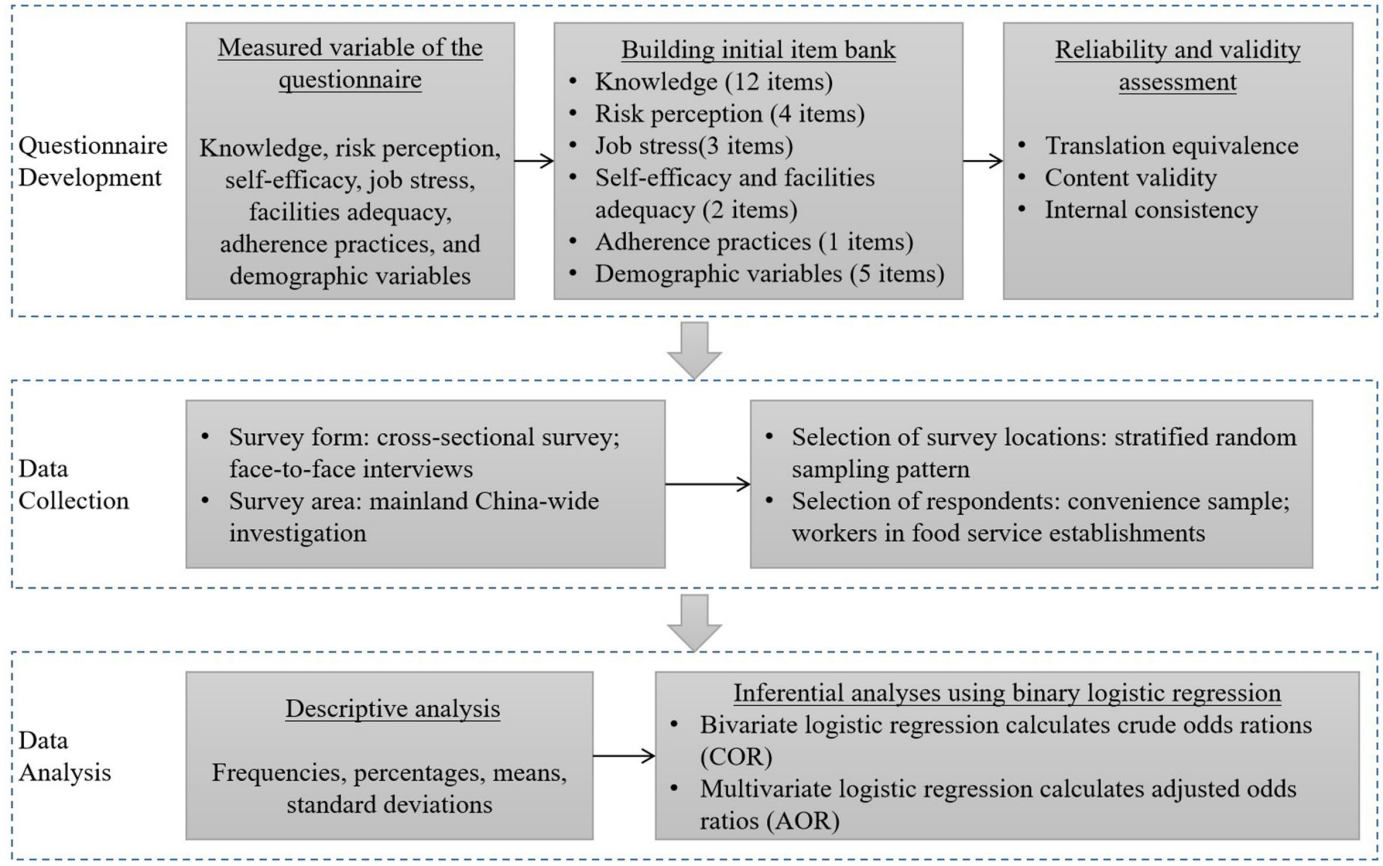
Research flow chart.

To obtain data to examine food worker adherence to WHO-initiated hygiene practices and its drivers, a cross-sectional survey using credible questionnaires was conducted Figure 1 details the questionnaire design and data collection process and data analysis methods.

### 2.1. Study design and data collection

A mainland China-wide investigation was carried out for the purpose of collecting representative data. A stratified random sampling pattern was adopted to select specific survey locations: one randomly selected province in eastern, central, and western regions of China, followed by two randomly selected cities in each province to conduct the survey. Respondents in each city were employed using a convenience sample. Food workers of each city’s food service establishments, such as restaurants, street vendors, and food retail stores, were invited to deliver the questionnaires, which were completed in the form of face-to-face interviews. The investigators explained the purpose of the survey, ensured anonymity and confidentiality, and obtained the consent of the respondents. After completing the questionnaire, a gift worth 15 RMB (approximately $2.2) was returned to the respondent. The completion time for each questionnaire was ranging from 15 to 30 min.

The survey was conducted from July to September 2022, which is during COVID-19. A total of 900 questionnaires, 150 per city, were distributed in six cities in mainland China, including Jining and Qingdao in Shandong Province in the eastern region, Zhumadian and Pingdingshan in Henan Province in the central region, and Chengdu and Panzhihua in Sichuan Province in the western region. A total of 762 questionnaires were recalled, with a response rate of 84.67% (762/900); after excluding the questionnaires containing missing values, 609 valid questionnaires were obtained, with a validate rate of 79.92% (609/762).

Ethical approval is approved by the Department of Marketing, the College of Business and Management, Jilin University.

### 2.2. Questionnaire development

The questionnaire was designed by reviewing the relevant literature and WHO guidelines (23), which consisted of 3 sections. The first section measures food workers’ knowledge of the hygiene practices recommended by the WHO guidelines with 12 items, which referred to studies by Wang et al. (6) and WHO guidelines (7). The knowledge items were surveyed using multiple choices, with at least one correct choice for each item. The correct choices are set with reference to the WHO guidelines. The principles of WHO-initiated hygiene practices include proper hand hygiene, good respiratory hygiene, frequent cleaning/sanitizing of work surfaces and contact points, and avoiding close contact with people showing symptoms of respiratory illness. An introduction to the WHO-initiated hygiene practices is provided after the knowledge section, followed by the second section, which consists of measuring food workers’ risk perception, self-efficacy, job stress, facilities adequacy, and adherence practices evaluated using a 5-point Likert scales. Except for the questionnaire measuring practices, all other variables were measured by a multiple-item format. Among them, risk perception was measured by 4 items originated from Jeong and Ham (24), job stress by 3 items originated from Bani-Melhem et al. (25), and self-efficacy and facilities adequacy by 2 items originated from Vassallo et al. (26) and de Andrade et al. (27), respectively. Worker adherence practices were measured using a frequency scale with one item. In order to avoid bias associated with self-reported and social desirability and to stimulate participants to state their actual practices, items were constructed according to Ajzen’s (28) principles of behavior scale development. The third section investigates the demographic variables of the respondents, including gender, age, educational level, income, and place of residence.

After the initial item set was developed, we used multiple methods to ensure the reliability and validity of the questionnaire. First, as all the items were adapted from the English document, two bilingual researchers in English and Chinese were employed to translate and back-translate the items to ensure translation equivalence (29). Second, a panel of seven experts, including two professors, two assistant professors, and three PhD candidates with research areas in food safety or behavioral sciences, was established to evaluate the content validity. The evaluation included consistency of items with the concept of variables, overlap of items, ambiguity of statements, understanding and reasonableness of items, and suitability of items to the Chinese context. The initial items were revised on the basis of the expert panel’s recommendations. Third, we conducted a pre-survey among 30 food workers, and we again modified those items that were difficult to understand or unclearly stated following the comments of these participants. Moreover, for variables measured by multiple items, Cronbach α coefficients were calculated to estimate the internal consistency. The Cronbach α coefficients of all variables exceeded the threshold value of 0.7, indicating good internal consistency (30). Following the above procedure, a questionnaire with 29 items was developed, of which the first section consists of 12 items measuring knowledge, the second section consists of 12 items measuring risk perception, self-efficacy, job stress, facilities adequacy, and adherence practices, respectively, and the third section consists of 5 items measuring demographic variables.

### 2.3. Data coding and analysis

For knowledge items, 1 score was recorded if the respondent did not choose the wrong option and 0 for the others. The 5-Likert scale encoding range is from 1 to 5. For variables measured by multiple items, a composite index was obtained from the mean to indicate the score of each variable. Using Bloom’s cutoff, respondents were categorized as good practices when their adherence practices score exceeded 3 (more than 60% on a 5-point Likert scale) and poor practices otherwise (31).

IBM SPSS software, version 22.0 was used to perform data analysis which included descriptive and inferential analyses. Descriptive analysis included the calculation of frequencies, percentages, means, standard deviations, and 95% confidence intervals (95% CI). Binary logistic regression with a stepwise-backward approach was employed to uncover the drivers that had significant effects on food workers’ adherence to WHO-initiated hygiene practices. Factors with *p* < 0.25 in bivariate logistic regression were entered in multivariate logistic regression (5). Crude (COR) and adjusted odds ratios (AOR) along with 95% CI were calculated to evaluate the effect strength. The fit of the model was tested using the Hosmer-Lemeshow goodness-of-fit test. Pseudo-*R^2^* (Nagelkerke *R^2^*) is presented to indicate the amount of variation in the dependent variable explained by the independent variable. A *p* < 0.05 was considered statistically significant.

## 3. Results

### 3.1. Information about the participants

Of the 609 final valid questionnaires, the majority of food workers were female at 65.4% (95% CI: 61.4, 69.1%). Nearly half of the respondents (48.9%; 95% CI: 44.8, 52.7%) were aged between 35 and 54 years. The education level of the food workers surveyed was generally low, with over 80% having a senior high school education or less. Food workers are also generally not well paid, with nearly half of the respondents (49.9%; 95% CI: 46.0, 54.0%) earning RMB 3,000 (around $436) or less per month. Respondents with residence in urban areas outnumbered those in rural areas, with 57.0% (95%CI: 52.9, 61.2%) and 43.0% (95% CI: 38.8, 47.1%), respectively. Details of the sample profile are reported in Table 1.

**Table 1:** Socio-demographic characteristics of food workers (*N* = 609).

Characteristics	Categories	Frequency	Percentage	95% CI
Gender				
	*Male*	211	34.6%	(30.9, 38.6%)
	*Female*	398	65.4%	(61.4, 69.1%)
Age in year				
	*18 ~ 34*	230	37.8%	(34.2, 41.7%)
	*35 ~ 54*	298	48.9%	(44.8, 52.7%)
	*≥55*	81	13.3%	(10.5, 16.1%)
Educational level				
	*Junior high school and below*	347	57.0%	(53.2, 60.9%)
	*Senior High School*	156	25.6%	(22.2, 28.9%)
	*College and above*	106	17.4%	(14.3, 20.5%)
Monthly income				
	*3,000 RMB and below*	304	49.9%	(46.0, 54.0%)
	*3,001 ~ 5,000 RMB*	182	29.9%	(26.3, 33.5%)
	*5,000 RMB above*	123	20.2%	(16.9, 23.6%)
Place of residence				
	*Urban*	262	43.0%	(38.8, 47.1%)
	*Rural*	347	57.0%	(52.9, 61.2%)

### 3.2. Personal and environmental factors and adherence practices of food workers

The survey results of the food workers’ knowledge about WHO-initiated hygiene practices showed the average correct rate was only 51.09% (95% CI: 49.25, 52.75%), which is a relatively low correct rate. The item with the highest correct rate was about the correct procedure for cleaning kitchen surfaces at 78.8% (95% CI: 75.4, 81.9%), followed by the operation after hand contact with the face at 71.8% (95% CI: 68.1, 75.4%). The item with the lowest correct rate was how long hand washing should last at 32.3% (95% CI: 28.9, 36.1%), followed by optimal way to dry hands after washing at 39.2% (35.5, 43.3%). The survey results for knowledge are presented in Table 2.

**Table 2:** Descriptive statistics on food workers’ knowledge in terms of WHO guidelines (*N* = 609).

Items	Correct response		
	Frequency	Percentage	95% CI
1. Is washing hands after handling raw food an effective way to reduce the spread of pathogens?	310	50.9%	(47.0, 54.8%)
2. Is it enough to wash your hands with only water?	287	47.1%	(43.2, 50.9%)
3. Which is the correct procedure for cleaning kitchen surfaces?	480	78.8%	(75.4, 81.9%)
4. Is it right to wash your hands regularly with alcohol-based hand sanitizer?	315	51.7%	(47.6, 56.2%)
5. When coughing or sneezing, should one cover the mouth and nose with a bent elbow or tissue?	293	48.1%	(44.3, 52.2%)
6. If you wash your hands well with water, is it unnecessary to use soap?	317	52.1%	(48.1, 55.8%)
7. Does the washing hands poorly cause disease?	408	67.0%	(63.2, 70.8%)
8. How long should hand washing take?	197	32.3%	(28.9, 36.1%)
9. Should you wash your hands after touching your face?	437	71.8%	(68.1, 75.4%)
10. Is there a need to wash hands with soap after sneezing?	382	62.7%	(58.9, 66.5%)
11. Which is the optimal manner to dry your hands after washing them?	239	39.2%	(35.5, 43.3%)
12. Should one avoid contact with people who sneeze or cough?	379	62.2%	(58.5, 66.0%)
Total Knowledge: Min. = 0; Max. = 11; Mean. = 6.13, 95 CI% = (5.93, 6.33); SD = 2.62; Average correct rate = 51.09%, 95 CI% = (49.25, 52.75%)			

For the other two variables in the cognitive dimension of the personal factor, the mean scores for risk perception were lower than the self-efficacy ones, at 3.37 and 3.67, respectively, with a range of 1 to 5 (see Table 3). Notably, for risk perception, the food workers rated the non-adherence to hygiene practices as most likely to reduce customer satisfaction (mean = 3.57; 95% CI: 3.49, 3.66), followed by the possibility of spreading COVID-19 (mean = 3.42; 95% CI: 3.33, 3.51), and the lowest likelihood of perceiving a negative impact on their own lives (mean = 3.20; 95% CI: 3.12, 3.29). For the affective dimension of the personal factor, the mean score for job stress was 3.11 (95% CI: 3.04, 3.18).

**Table 3:** Descriptive statistics on risk perception, self-efficacy, job stress, facilities adequacy, and practices (*N* = 609).

Items	Min.	Max.	Mean.	SD	95%CI
*Risk perception (Cronbach α = 0.859)*	1	5	3.37	0.911	(3.29, 3.44)
If I do not adhere to the above hygiene practices,					
1. then the risk of spreading COVID-19 will be high.	1	5	3.42	1.048	(3.33, 3.51)
2. then there is a risk of causing foodborne illness.	1	5	3.27	1.072	(3.18, 3.36)
3. then there is a possibility that my life would be adversely affected.	1	5	3.20	1.129	(3.12, 3.29)
4. then customer satisfaction may be reduced.	1	5	3.57	1.095	(3.49, 3.66)
*Self-efficacy (Cronbach α = 0.727)*	1	5	3.67	0.866	(3.60, 3.74)
1. Adherence to the above hygiene practices is easy for me.	1	5	3.70	0.980	(3.62, 3.77)
2. Compliance with the above hygiene practices is under my control.	1	5	3.65	0.974	(3.56, 3.73)
*Job stress (Cronbach α = 0.788)*	1	5	3.11	0.872	(3.04, 3.18)
1. I have too much work and too little time to do it.	1	5	3.21	1.047	(3.13, 3.29)
2. My work affects me more than it should.	1	5	3.22	1.031	(3.13, 3.30)
3. At the end of the workday, I felt worn out.	1	5	2.89	1.044	(2.81, 2.98)
*Facilities adequacy (Cronbach α = 0.769)*	1	5	3.92	0.739	(3.86, 3.98)
1. The equipment items required to perform the above hygiene practices are readily available in my workplace.	1	5	3.95	0.820	(3.89, 4.02)
2. The facilities at my workplace are of sufficient quality to perform the above hygienic practices at all times.	1	5	3.89	0.819	(3.83, 3.96)
*Practices*					
1. In the past week, how often did you adhere to the above hygiene practices?	1	5	3.46	0.912	(3.39, 3.54)

Facilities adequacy, as the environmental factor of concern, had an mean score of 3.92 (95% CI: 3.86, 3.98), implying that the availability of equipment to perform hygiene practices was generally perceived to be high by the food workers surveyed.

**Figure 2:**
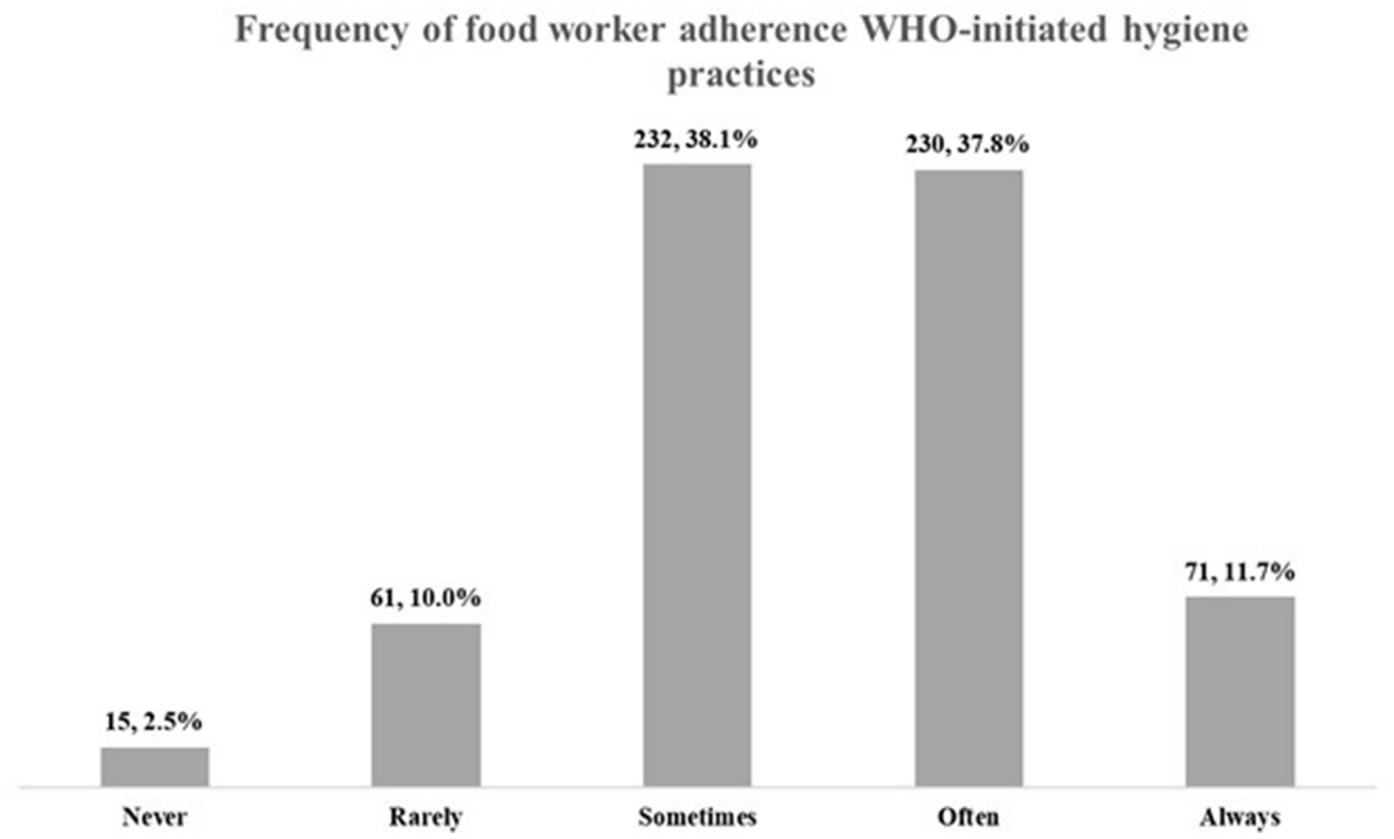
Frequency of adherence practices reported by surveyed food workers (*n* = 609).

The mean score for food worker adherence practices was 3.46 (95% CI: 3.39, 3.54), with “sometimes” and “often” being the most frequent responses, chosen by 232 (38.1%) and 230 (37.8%) respondents, respectively (see Figure 2). Only 71 workers, accounting for 11.7%, chose “always” to adhere to WHO-initiated hygiene practices. After being classified by Bloom’s cutoff approach, 308 and 301 workers were “poor practices” and “good practices,” respectively.

### 3.3. Factors associated with adherence to who-initiated hygiene practices

Binary logistic regression was performed separately for each of the personal, environmental, and demographic variables and adherence practices, and it was found that all variables met the threshold of *p* < 0.25, and thus all were entered into multivariate logistic regression to examine the association with the outcome variable. The Hosmer-Lemeshow goodness-of-fit statistical test is insignificant (*p* = 0.409), indicating that the final model is considered to have good fit. The final model explained 23.8% (Nagelkerke *R^2^* = 0.238) of the variance in food workers’ adherence to WHO-initiated hygiene practices and revealed that monthly income, place of residence, knowledge, self-efficacy, and risk perception were significant drivers (see Table 4).

**Table 4:** Determinants of food workers’ adherence to WHO guidelines (*N* = 609).

Variable	Poor practice	Good practice	COR (95%CI)	*p*-value	AOR (95%CI)	*p*-value
	*N* = 308	*N* = 301				
***Gender***						
Female	115(54.5%)^a^	96(45.5%)^a^	1		1	
Male	193(48.5%)^a^	205(51.5%)^a^	1.27(0.91, 1.78)	0.158	1.18(0.81, 1.73)	0.385
***Age in year***						
18 ~ 34	114(49.6%)^a^	116(50.4%)^a^	1	0.016	1	0.613
35 ~ 54	141(47.3%)^a^	157(52.7%)^a^	1.09(0.78, 1.54)	0.608	1.00(0.66, 1.52)	0.986
≥55	53(65.4%)^a^	28(34.6%)^a^	0.52(0.31, 0.88)	0.015	0.78(0.40, 1.41)	0.377
***Educational level***						
Junior high school and below	178(51.3%)^a^	169(48.7%)^a^	1	0.233	1	0.131
Senior high school	84(53.8%)^a^	72(46.2%)^a^	0.90(0.62, 1.32)	0.597	0.72(0.46, 1.12)	0.144
College and above	46(43.4%)^a^	60(55.6%)^a^	1.37(0.89, 2.13)	0.155	1.28(0.74, 2.22)	0.380
***Monthly income***						
3,000 RMB and below	147(48.4%)^a^	157(51.6)% ^a^	1	0.436	1	<0.001
3,001 ~ 5,000 RMB	99(54.4%)^a^	83(45.6%)^a^	0.79(0.54, 1.14)	0.198	0.40(0.25, 0.62)	<0.001
5,000 RMB above	62(50.4%)^a^	61(49.6%)^a^	0.92(0.61, 1.40)	0.701	0.72(0.43, 1.21)	0.213
***Place of residence***						
Urban	104(39.7%)^a^	158(60.3%)^a^	1		1	
Rural	204(58.8%)^a^	143(41.2%)^a^	0.46(0.33, 0.64)	<0.001	0.44(0.30, 0.65)	<0.001
***Knowledge***	5.59(2.51)^b^	6.68(2.62)^b^	1.18(1.11, 1.26)	<0.001	1.09(1.01, 1.18)	0.022
***Self-efficacy***	3.36(0.90)^b^	3.99(0.70)^b^	2.64(2.10, 3.32)	<0.001	2.51(1.91, 3.28)	<0.001
***Risk perception***	3.20(0.87)^b^	3.54(0.92)^b^	1.52(1.27, 1.83)	<0.001	1.28(1.03, 1.60)	0.028
***Job stress***	3.06(0.88)^b^	3.15(0.86)^b^	1.13(0.94, 1.36)	0.192	1.01(0.81, 1.26)	0.951
***Facilities adequacy***	3.79(0.72)^b^	4.06(0.73)^b^	1.66(1.32, 2.09)	<0.001	1.08(0.82, 1.43)	0.591

a The percentages in parentheses are calculated by row, based on the available information summed to 100 percent.

b The figures outside the parentheses refer to the mean and the figures inside the parentheses refer to the SD.

COR refers to crude odds ratio; AOR refers to adjusted odd ratio; CI refers to confidence interval.

In terms of income, food workers earning 3,001 to 5,000 RMB per month were the least likely to adhere to hygienic practices, 0.40 (AOR: 0.40; 95% CI: 0.25, 0.62) times as likely as those earning 3,000 RMB per month and below to adhere. Food workers with rural residence were 0.44 (AOR: 0.44; 95% CI: 0.30, 0.65) times as likely to adhere to the practices compared with their urban counterparts. The cognitive dimensions of personal factors both have a significant positive influence on adherence practices, with the largest effect being self-efficacy (AOR: 2.51; 95% CI: 1.91, 3.28), followed by risk perception (AOR: 1.28; 95% CI: 1.03, 1.60) and knowledge (AOR: 1.09; 95% CI: 1.01, 1.18). The effects of job stress (AOR: 1.01; 95% CI: 0.81, 1.26), representing the affective dimension, and facilities adequacy (AOR: 1.08; 95% CI: 0.82, 1.43), representing the environmental factors, were insignificant.

## 4. Discussion

Adherence to WHO-initiated hygiene practices by food workers in food service establishments contributed positively to both limiting COVID-19 transmission and reducing the incidence of foodborne illness (7, 8), but the drivers of adherence practices are uncertain. Being the pioneer in employing social cognitive theory to analyze food workers’ adherence to hygiene practices, this study used three components to identify drivers of practices in terms of personal, environmental, and demographic variables. By analyzing the 609 valid responses collected *via* the credible questionnaire, knowledge, risk perception, self-efficacy, monthly income, and place of residence were confirmed to have significant roles in adherence practices, which provides direction for tailoring theory-guided intervention programs.

Our survey revealed that near half of the knowledge of hygiene practices is poorly understood by food workers, which implies that the knowledge of food handling possessed by professionals who handle food is significantly inadequate. This result is similar to the results of Habte et al. (5) survey on food handlers’ knowledge in Ethiopia (mean correct rate = 58.4%) and Olaimat et al. (32) survey on food handlers’ knowledge in Jordan, but lower than the results of Ferreira et al. (13) survey on food workers in Brazil (mean correct rate = 72.2%). Two possible reasons for the inconsistency are that the knowledge measurement items differ, and the other is that Ferreira et al. (13) study only targeted food workers in public school food services, who, since food safety in schools is of great concern, may have more training. Moreover, knowledge was also identified as a significant promoter of worker adherence to hygiene practices, which is consistent with the outcome of many previous studies, such as Habte et al. (5), Akabanda et al. (10), Sani and Siow (12). It should be noted, however, that while knowledge has a significant positive effect, the effect magnitude is not strong that is significantly lower than the self-efficacy and risk perception ones in the cognitive dimension, implying that the knowledge–behavior gap still remains among food workers (33). This also suggests that factors other than knowledge should receive more attention in the intervention.

The mean score for risk perception is only slightly above neutral, indicating that workers do not view the likelihood of not adhering to hygiene practices causing a wide range of adverse outcomes as high. This belief would lead workers to underestimate the possibility of putting themselves or others at risk in this way, presenting an error in perception and control of the activities they handle (34). Optimism bias was also found to be common in how workers viewed food handling practices (34, 35). In addition, respondents gave different ratings to various dimensions of risk perception, which may be caused by their valuing consumer satisfaction and their fear of COVID-19. The increasing number of cases and deaths associated with the COVID-19 pandemic has raised a palpable fear among the public (36). Among the theories that explain health-promoting behavior, such as the Health Belief Model (37) and the Protective Motivation Theory (38), it is claimed that increasing the risk perception motivates individuals to engage in protective behavior. This study also points to risk perception as a significant driver of adherence to hygiene practices, which signifies that the formulation of intervention strategies should include components that shape the correct risk beliefs of food workers. Messages aimed at raising public awareness of health and safety risks have been demonstrated to have the potential to promote the adoption of risk-averse and health-related behaviors by the public (39).

Out of the personal and environmental factors focused on in this study, self-efficacy ranks as the most effective motivator of worker adherence to hygiene practices. A systematic review by Young et al. (40) found that self-efficacy has been confirmed by some studies to contribute to predicting the likelihood of food handlers engaging in correct food handling practices. Wang (41) also pointed out that self-efficacy predicted a large proportion of variance in the food safety practices of school food service staff. Self-efficacy has also been recommended to be included in the framework of interventions for food handling practices (42). Our study is also consistent with these findings and proposes that stronger self-efficacy of food workers to carry out hygiene practices may be more effective than other interventions.

What is interesting to note is that contrary to the cognitive dimension, job stress, representing the affective dimension, is not a significant motivator. This suggests, on the one hand, that the cognitive dimension plays a more pivotal role in shaping workers’ adherence to hygiene practices. There are, on the other hand, inconsistent results from previous analyses of the effect of job stress on the decision-making of food service workers (25, 27), which suggests that job stress may not have a direct effect on behavioral decisions, but rather an indirect one (43). Hence, the effect of job stress on adherence practices needs to be further explored. With other words, there is no direct effect of job stress, but there may be an indirect effect or a co-effect with other variables.

Facilities adequacy, as the environmental factor of interest, has a mixed impact on adherence practices. This is notable in that workers rated the facilities adequacy at a remarkably high level, which means that the food service establishments surveyed have the capacity to provide the equipment needed to adherence practices. The significant COR coefficient and the non-significant AOR coefficient indicate a significant positive correlation between facilities adequacy and adherence practices, but this correlation is moderated by other factors, such as personal factors and demographic variables. While some previous studies have shown that facilities adequacy imposes significant influence on food worker practices (14, 27), this research’s results imply that these findings need to be taken with a pinch of salt with more factors being controlled for to separate out the net effect of facilities adequacy. Under the assumptions of stimulus-organism-response framework (S-O-R), the impact of the external environment (stimulus) on individual psychological or behavioral change (response) is *via* the person’s internal decision-making processes (organism) (44). The findings of this study also mean that it is a more feasible direction for the future to analyses the effects of facilities adequacy with S-O-R.

Income and place of residence in the demographic variables are two significant factors contributing to workers’ adherence practices. Some studies have also confirmed the significant effect of these two variables on the food hygiene practices (3, 45, 46). Food workers with a household income of RMB 3001–5,000 and those living in rural settings performed significantly less frequently than their counterparts in adopting adherence practices, suggesting that these groups are high risk groups for not implementing WHO-initiated hygiene practices that need to be prioritized for intervention programs. Moreover, it is interesting to note that the lowest-income worker group (3,000 RMB and below) reported the greatest adherence practices. The reason for this may be that the lower income groups have a greater perception of uncertainty (47) and fear of losing their jobs, hence they are more inclined to comply with the guidelines.

### 4.1. Policy implication

By examining the effects of personal, environmental, and demographic variables on food worker adherence practices in the context of SCT, this study provides guidance for determining priority intervention information and populations for promoting the adoption of WHO-initiated hygiene practices. In view of the significant effect of the cognitive dimension of the personal factors, intervention design should devote more attention to shaping the proper perceptions of workers regarding hygiene practices. Of these, self-efficacy, due to its most powerful role, should be given the most priority. Low self-efficacy can cause workers to perceive difficulties in performing hygiene practices and low confidence, which may limit volitional control and lead to the non-adherence to these practices (48). When conducting worker training, participants may be asked to write down future plans for their practice, set goals for adherence to hygiene measures and create a commitment to these goals in the form of a signature, all of which are effective ways to increase self-efficacy (49, 50). Adopting simulation training to deal with potential barriers to practice also helps to increase worker’ sense of confidence (9).

Risk perception and knowledge, the other two sub-components of the cognitive dimension, are also priority intervention information. Notably, while knowledge has a significant positive effect, the weak effect strength means that the treatment effect of increasing workers’ knowledge of hygiene practices alone is limited. In order to improve adherence by workers more effectively, it is essential to spread information about the various adverse outcomes that may result from non-adherence to hygiene practices, alongside the introduction of knowledge about WHO-initiated hygiene practices, to develop a correct risk perception among workers. Previous research suggests that different expressions of the same information produced significantly different intervention effects, and the framing effect can be taken into account when designing intervention materials (51, 52).

Intervention programs should be tailored to different populations with different degrees of priority. Workers with incomes from 3,001 to 5,000 RMB and those living in rural areas should be given more attention as these groups show less adherence than their counterparts. Designed intervention strategies tailored to different populations are also considered to lead to more effective outputs (9, 50).

### 4.2. Research limitations

This study provides a comprehensive understanding of food workers’ adherence practices regarding WHO-initiated hygiene practices in the framework of SCT. However, some limitations remain. First, the investigation of adherence practices is in the form of self-report. Even though this is one of the most common methods to understand individual behavior, it has also been pointed out that there may be discrepancies between self-reported and true behavior (53, 54). This study has adopted multiple ways to ensure the validity of self-reported surveys, and future studies could also be conducted using observations. Second, statistical inferential analysis is predicated on cross-sectional data and what is identified is only linear interrelationships of variables, which may reduce the internal validity of the findings (55). Moreover, the design of this study is to represent the results at one point in time and does not reflect the future findings of food worker adherence practices and its determinants. Finally, SCT is an influential theory in explaining behavioral decision-making, but the determinants of worker adherence practices are interpreted in terms of knowledge, risk perception, and self-efficacy as cognitive dimension, work stress as affective dimension, and facility adequacy as environmental factors. It is encouraged that future research should explore other potentially influencing factors based on SCT in order to gain a better insight into worker adherence to hygiene practices.

## 5. Conclusion

Adherence to WHO-initiated hygienic practices by food workers is a two-birds-one-stone strategy to prevent the spread of COVID-19 and limit the occurrence of foodborne illness, but the determinants of motivating workers to adhere to the practices are unclear. In this study, we shed some light on the factors influencing workers’ adherence to hygiene practices from personal factors, environmental factors and demographic variables on the basis of SCT. Based on cross-sectional survey data obtained through face-to-face interviews in food service establishments in East, Central, and West China, our study indicates that the knowledge level of workers regarding WHO-initiated hygiene practices is insufficient, the frequency of adherence to hygiene practices is poor, and needs to be improved. Furthermore, three of the cognitive dimensions of the personal factors, including knowledge, risk perception, and self-efficacy, had significant positive effects on adherence practices. Of these, self-efficacy had the strongest effect. There are also significant differences in adherence practices under conditions of different income levels and places of residence. These insights provide evidence-based guidance in determining priority intervention information and population for improving food worker adherence to WHO-initiated hygiene practices.

## Data availability statement

The raw data supporting the conclusions of this article will be made available by the authors, without undue reservation.

## Ethics statement

The studies involving humans were approved by the Department of Marketing, the College of Business and Management, Jilin University. The studies were conducted in accordance with the local legislation and institutional requirements. The participants provided their written informed consent to participate in this study.

## Author contributions

MW: conceptualization, data curation, formal analysis, investigation, methodology, validation, writing – original draft, writing – review and editing. SG: resources and supervision. JT: investigation, supervision, and project administration. ZW: resources and supervision. XW: conceptualization, investigation, writing – review and editing, and funding acquisition. All authors contributed to the article and approved the submitted version.
